# Automated Method for Identification and Artery-Venous Classification of Vessel Trees in Retinal Vessel Networks

**DOI:** 10.1371/journal.pone.0088061

**Published:** 2014-02-12

**Authors:** Vinayak S. Joshi, Joseph M. Reinhardt, Mona K. Garvin, Michael D. Abramoff

**Affiliations:** 1 Department of Biomedical Engineering, University of Iowa, Iowa City, Iowa, United States of America; 2 Department of Electrical and Computer Engineering, University of Iowa, Iowa City, Iowa, United States of America; 3 The Center for the Prevention and Treatment of Visual Loss, Iowa City VA Health Care System, Iowa City, Iowa, United States of America; 4 Department of Ophthalmology and Visual Sciences, University of Iowa, Iowa City, Iowa, United States of America; 5 The Iowa City Veteran's Medical Center, Iowa City, Iowa, United States of America; University of Navarra, Spain

## Abstract

The separation of the retinal vessel network into distinct arterial and venous vessel trees is of high interest. We propose an automated method for identification and separation of retinal vessel trees in a retinal color image by converting a vessel segmentation image into a vessel segment map and identifying the individual vessel trees by graph search. Orientation, width, and intensity of each vessel segment are utilized to find the optimal graph of vessel segments. The separated vessel trees are labeled as primary vessel or branches. We utilize the separated vessel trees for arterial-venous (AV) classification, based on the color properties of the vessels in each tree graph. We applied our approach to a dataset of 50 fundus images from 50 subjects. The proposed method resulted in an accuracy of 91.44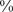 correctly classified vessel pixels as either artery or vein. The accuracy of correctly classified major vessel segments was 96.42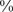.

## Introduction

Several automated techniques have been reported to quantify the changes in morphology of retinal vessels (width, tortuosity) indicative of retinal or cardiovascular diseases. Some of the techniques measure the vessel morphology as an average value representing the entire vessel network, e.g., average tortuosity [1]. However recently, vessel morphology measurement specific to arteries or veins was found to be associated with disease. For example, ‘plus’ disease in retinopathy of prematurity (ROP) may result in increase in arterial tortuosity relative to that of veins indicating the need for preventative treatment [2]. Arterial narrowing, venous dilatation, and resulting decrease in artery-to-venous width ratio (AVR) may predict the future occurrence of a stroke event or a myocardial infarct [3]. Unfortunately, the detection of minute changes in vessel width or tortuosity specific to arteries or veins may be difficult in a visual evaluation by an ophthalmologist or by a semi-automated method, which is laborious in clinical practice. Therefore, an automated identification and separation of individual vessel trees and the subsequent classification into arteries and veins is required for vessel specific morphology analysis [4].

There is a dearth of methods developed for retinal vessel tree separation and identification in color fundus images, which is an important step for a robust AV classification. As per our knowledge, the only method developed with this objective was reported by Lau et al., using an optimal forest search on segmented vessel trees given a set of constraints based on directional information [5]. The other method for retinal vessel separation, however not applicable to color fundus images, was reported by Vickerman et al., that is applicable to fluorescence angiogram images only where arteries are identified as they are filled with contrast before veins [4]. The contrast based separation is then propagated into the entire vessel network using morphologic and connectivity features of retinal vessels. A method proposed by Aylward et al. for intra-cranial vessel separation and identification is noteworthy [6], although not directly related to the proposed retinal vessel analysis. This method is applicable to three-dimensional computed tomography (CT) and magnetic resonance angiogram (MRA) volumes, and provides a graphical representation of a vessel network for identification of individual vessels known as spatial graphs, based on the analysis of branching topology and vessel segment paths between two branchings.

Several artery-venous (AV) classification methods have been proposed based on the analysis of localized vessel structure. Rothaus et al. proposed a semi-automatic constraint optimization approach based on artery-venous crossing properties and anatomic characteristics [7]. The central light reflex of retinal arteries was used as a distinguishing factor by Tramontan et al. [8]. Grisan et al. suggested a method based on division of the fundus into four regions of interest (ROI) and classification of blood vessels in each region using color properties of the vessels [9]. Vazquez et al. presented a clustering approach based on the feature sets obtained from retinal vessels [10]. A method by Kondermann et al. extracts a feature set from vessel profiles and local image intensities with respect to the vessel centerlines [11]. This method uses a support vector machine and neural networks for classification. A supervised classification approach was demonstrated by Niemeijer et al. in which the algorithm was trained on annotated vessel segments for feature extraction and the trained classifier was used to separate arteries from veins in a test dataset [3]. However, these automated methods allow AV classification constrained only to a region around the optic disc but not to the entire vessel network. [3], [9].

**Figure 1:**
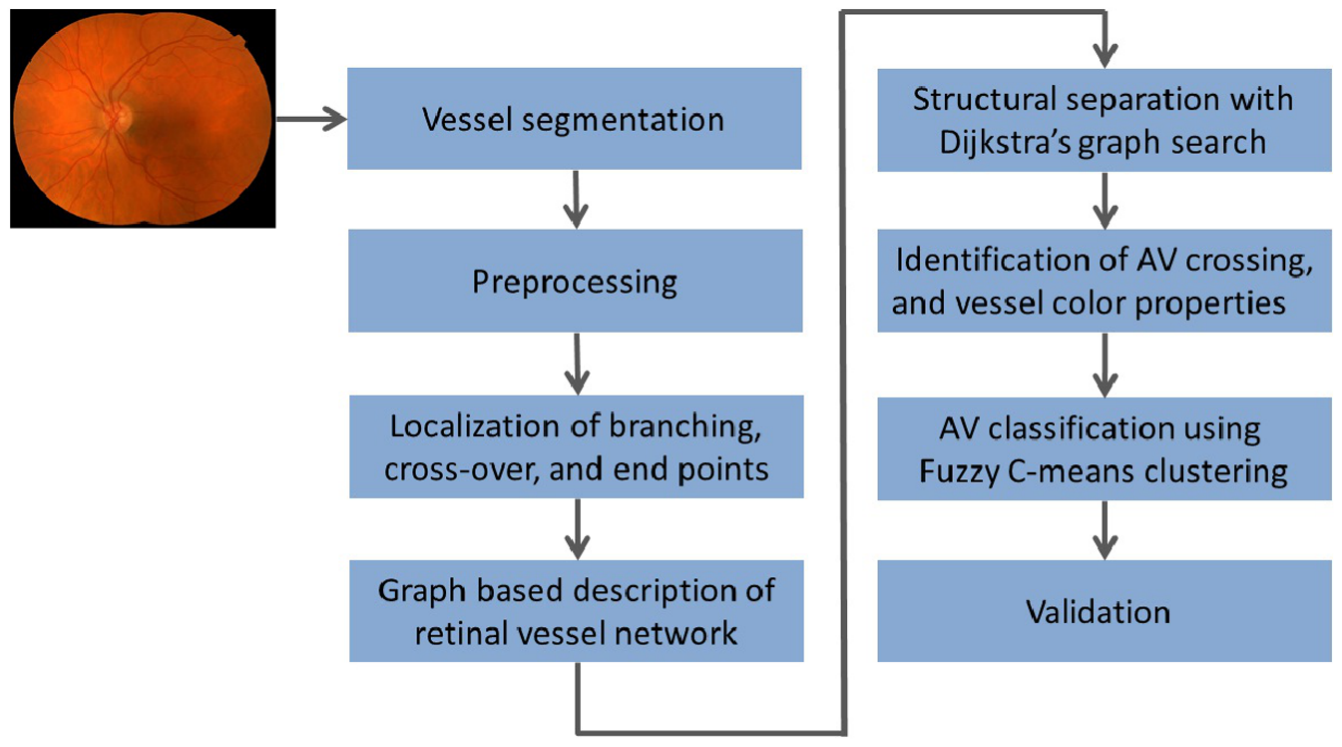
Overview of the steps in proposed method.

We introduce an automated method for structural mapping of retinal vessels by converting a vessel segmentation into a vessel segment map and identifying the vessel trees using graph search. Arterial-venous classification uses color features. We evaluated the method on a dataset of 50 color fundus images from 50 subjects and compared the results to manual annotation by an expert (MDA). An overview of our approach is described in (Fig. 1). Each of the steps is explained in detail as follows.

## Methods

### 0.1 Vessel segmentation and image preprocessing

**Figure 2:**
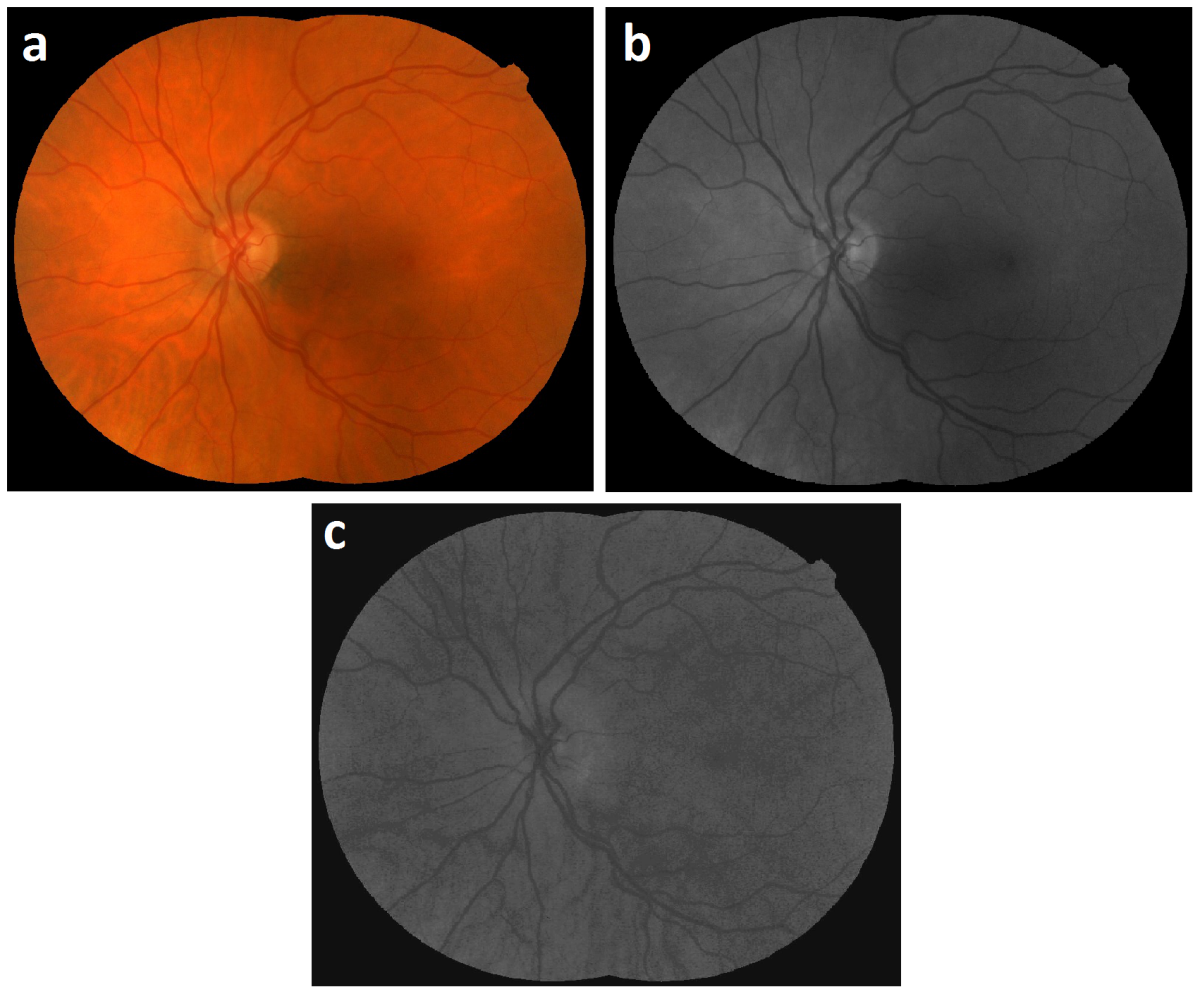
Image example: a) Two field fundus image after mosaicing b) Green channel image c) Hue channel image.

**Figure 3:**
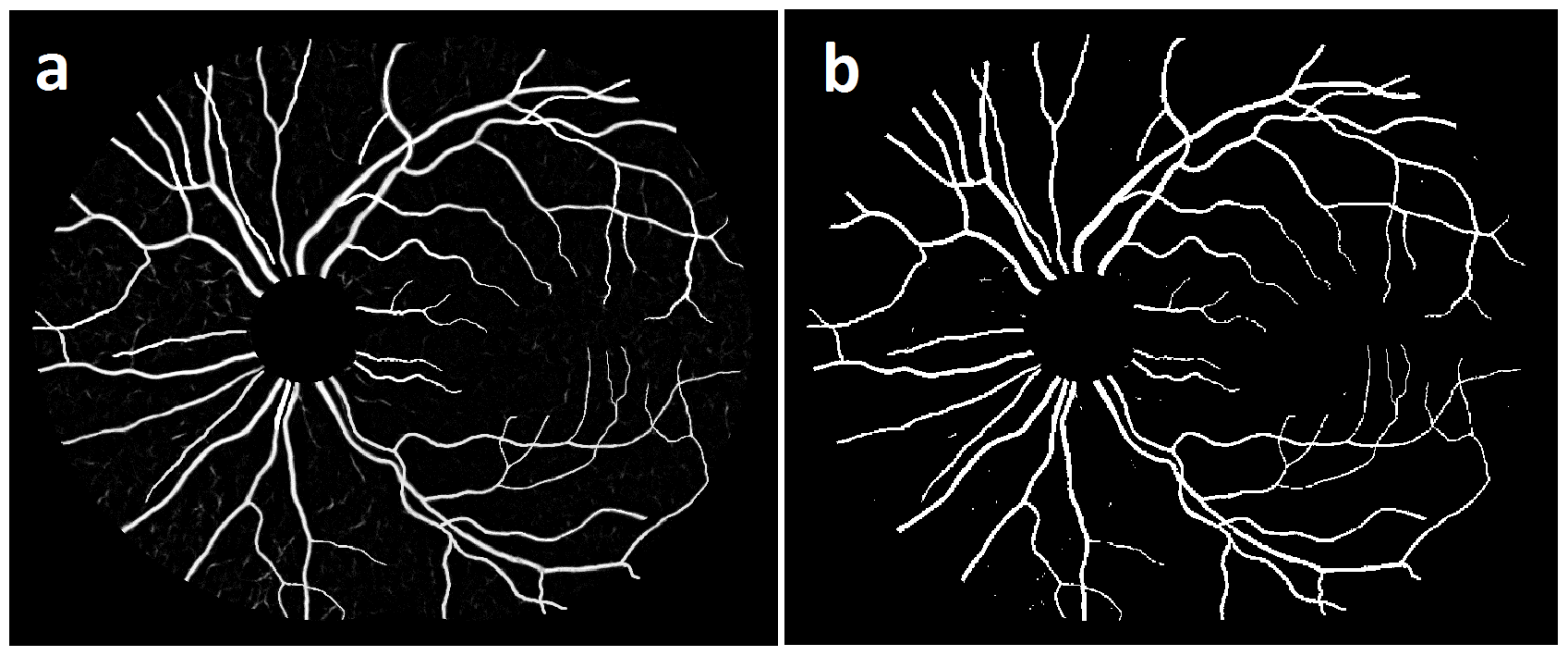
Image example: a) Vessel probability image b) Binary image.

Two-field fundus images of the same eye are registered by mosaicing [12] (Fig. 2(a)). The corresponding green channel image (Fig. 2(b)) and hue channel image (Fig. 2(c)) are shown. The retinal vessels are segmented using a previously developed approach that uses supervised pixel classification with a Gaussian filter set and classification by a k-nearest neighbor classifier [13]. The resulting vessel probability image represents the likelihood of each pixel belonging to a vessel (Fig. 3(a)). The optic disc (OD) region is masked manually using a standard size mask to reduce ambiguities from the highly tortuous and intertwined vessel patterns at the OD region. In order to trace the vessel path and obtain structural mapping, a connected binary vessel image is required which may be obtained using a vessel reconnection algorithm based on a graph search [14].

The binary vessel image (Fig. 3(b)) is generated from the vessel probability image using Otsu's thresholding method [15]. The Otsu threshold minimizes the intra-class variance for the foreground (vessel) and the background (non-vessel region) classes. Next, the vessel skeleton is obtained by applying mathematical morphology reducing the vessel to a centerline of single pixel width [16].

### 0.2 Localization of branch points, crossing points and end points

**Figure 4:**
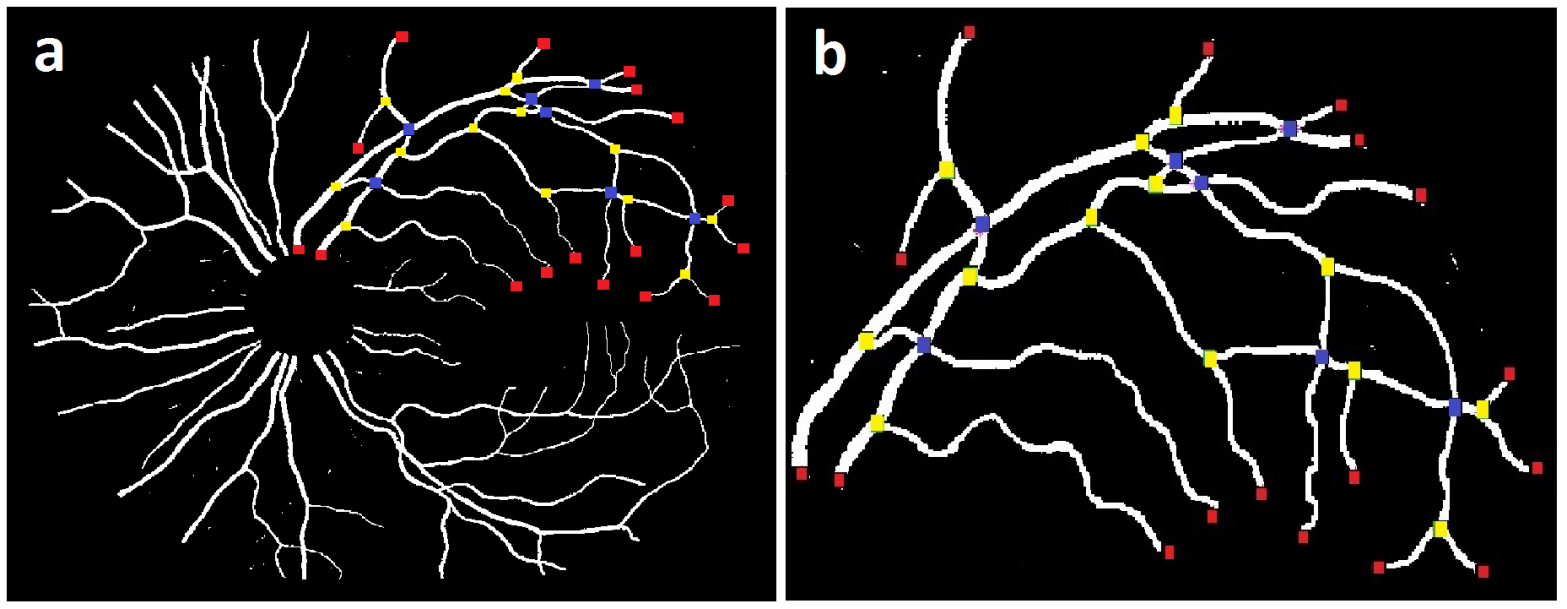
Image example: a) Vessel network b) Vessel tree [Vessel width is enlarged for visualization].

In order to represent the vessel structure in terms of a graph, the vessel skeletons have to be converted into vessel segments separated by interruptions at the branch- and crossing points. Segment start and end positions are determined as follows. Each of the centerline pixels on the vessel skeleton is analyzed within its 3×3 neighborhood, and branch and crossing points are detected as centerline pixels with more than 2 neighbors. The detection of vessel end points is required for the graph search and they are determined as the centerline pixels with only one neighbor. The Fig. 4(a) (vessel network), and Fig. 4(b) (vessel tree), show the end points (red), branching points (yellow), and crossing points (blue).

### 0.3 Graph based description of the retinal vessel network

#### 0.3.1 Graph structure

**Figure 5:**
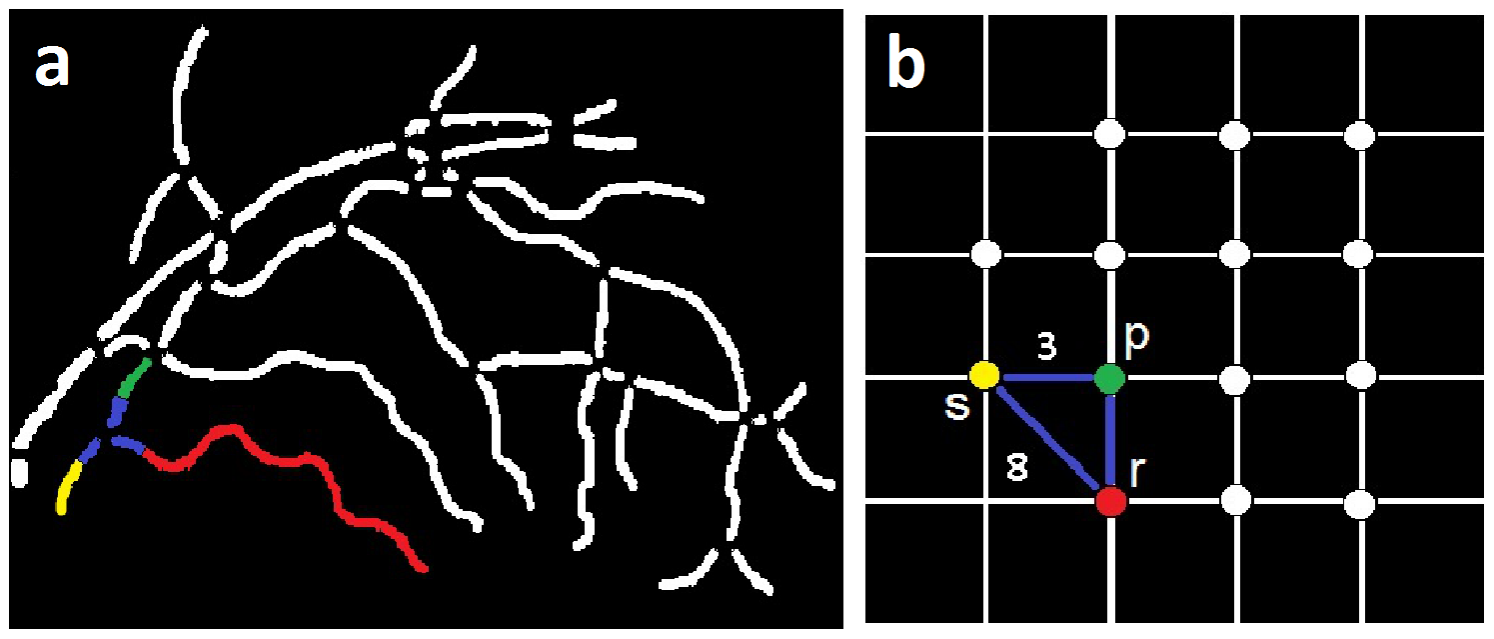
Graph based description: a) Vessel segment map [Width is enlarged for visualization] b) Representative graph structure.

In order to construct a graph, the vessel segment map (Fig. 5(a)) is obtained by removing branch and crossing points on vessels in a binary image (Fig. 3(b)), resulting in a group of disconnected vessel segments representing a vessel tree.

A vessel consists of a number of smaller vessel segments linked together [17]. Segment to segment characteristics within a single vessel, such as orientation, width, and intensity are expected to be similar [18], in the absence of noise. The inter-segment orientation angles follow a smooth continuous variation. Adjacent vessel segments exhibit fine continuous variation in widths, with some exceptions such as microaneurysms and vessel beading. Similarly, there is a gradual intensity transition between the neighboring segments.

A vessel subtree is identified by selecting a group of segments from the vessel segment map (Fig. 5(a)), based on the similarity between these segments. Three features are used 1) segment orientation, 2) segment width, and c) segment intensity (in the green channel), and these are the costs associated with each segment: segment orientation cost, segment width cost, and segment intensity cost. The features are measured at the end regions of each vessel segment, with skeletal length of 15 pixels from each end. Specifically, orientation is expressed as the angle (in radians) the segment end region makes with the positive direction of X-axis, a measurement between [0,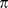]. The width (in pixel) is measured as a median value of 15 measurements of diametric length between the vessel edges, and passing through the skeleton pixels of the end region. The intensity is measured as a median value of green channel pixel intensities at the segment end region. The median value of the width and that of the intensity measured for each vessel segment of the vessel tree, are normalized by the respective maximum values obtained across that vessel tree. Although each segment is associated with three costs, at any time during the graph search, only one of the three costs is assigned to the segment, as described later.

To convert the vessel segment map (Fig. 5(a)) into a connected graph structure, connecting neighboring vessel segments are identified using branch and crossing-point information. In the derived graph structure 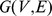 (Fig. 5(b)), nodes 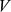 represent the corresponding vessel segments from the vessel segment map, and each edge 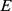 connecting any two nodes, represents the costs with respect to absolute difference in orientation (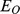), absolute difference in width (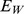), and absolute difference in intensity (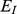), at the end regions of two vessel segments represented by two nodes. At any instant during the graph search, only one of the three difference costs is assigned to the edge, in a hierarchy as explained in algorithm in Table 1. The graph edges are initialized with ‘
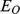
’ due to a high robustness in ‘orientation measurement’ irrespective of image resolution and image noise; however, the edges may later be assigned with 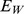 or 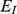 during a hierarchical step change (Algorithm: Table 1). The scales for width and intensity measurement units are matched with that for the orientation measurement unit, to prevent a scale bias in the graph search.

**Table 1:** Algorithm 1: Structural mapping using Dijkstra's graph search.

1:	**Input:** Graph ‘ 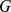 ’, with any pair of nodes a and b 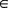 ‘ 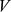 ’, and edge ‘ 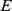 ’ in form 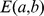 = 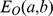 or 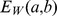 or 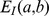
2:	**Output:** Nodes describing the minimum edge cost path: True vessel
3:	
4:	**for** i = each end point node 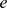 **do**
5:	S = set of explored nodes in 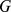 : Initially S = 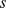 , Q = set of unexplored nodes in 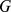
6:	For any node u, dist[u] = minimum path cost from 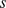 to u, previous[u] = parent node to u
7:	**while** u 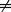 i: **do**
8:	u = node in Q with at least one edge to S, and smallest dist[]; remove u from Q, add u to S
9:	**for** each neighbor v of u: **do**
10:	Initialize 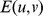 = 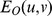
11:	**for** each neighbor v′ ( 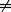 v) of u: **do**
12:	**Comment:** Compare width differences, if 2 orientation differences are equal
13:	**if** [ 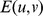 = = 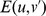 ] **then**
14:	Initialize 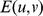 = 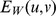 and 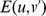 = 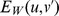
15:	**Comment:** Compare intensity differences, if 2 width differences are equal
16:	**if** [ 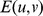 = = 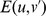 ] **then**
17:	Initialize 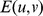 = 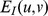 and 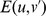 = 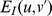
18:	**if** dist[v]>dist[u]+ 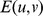 **then**
19:	dist[v] : = dist[u]+ 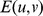 and previous[v] : = u
20:	Store path from 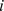 to 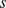 = previous[] and 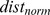 [i] = dist[i]/length(previous[])
21:	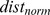 [e′] = min( 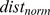 [e])
22:	True vessel path = previous[]; For a path from 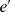 to 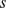

In fig. 5(a) three vessel segments are colored in yellow, green, and red as an example. The end regions connecting the three segments are marked in blue. The corresponding graph structure (Fig. 5(b)) shows a seed node ‘
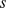
’ in yellow, identified as the node representing the root vessel segment in a vessel tree (a vessel segment containing an end point nearest to the circumference of the circle masking the optic disc; marked yellow in (Fig. 5(a))). Two other nodes ‘
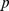
’ (green), and ‘
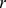
’ (red) are marked in respective colors representing the vessel segments. The edge (marked in blue) connecting any two nodes (e.g., ‘
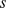
’ and ‘
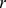
’) represents the three cost differences associated with two respective vessel segments. For an illustration, the orientation of yellow segment is more similar to that of green segment than the red segment (Fig. 5(a)). Therefore, the orientation difference cost of the edge between yellow and green node (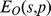 = 3) is lower compared to the orientation difference cost of the edge between yellow and red node (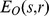 = 8) (Figure not to scale). A retinal vessel or its branches in a vessel tree do not form a cycle, i.e., do not cross themselves [7], and thus the vessel segments always follow a unidirectional vessel course starting at a root segment and ending at the end point segments, i.e., vessel segments containing the end points (marked red in (Fig. 4(b))). Therefore, while determining the orientation difference cost (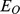) between two connecting neighboring vessel segments, their orientations with respect to the positive X-axis as well as their directions of following the vessel course are taken into consideration.

#### 0.3.2 Dijkstra's graph search

Dijkstra's algorithm is utilized to identify a vessel subtree. Equation 1 explains the operation of Dijkstra's algorithm which searches for a minimum edge cost path ‘dist[t]’ that connects any node ‘
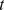
’ with the seed node ‘
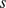
’ by minimizing the sum of edge costs ‘
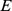
’ between intermediate nodes ‘
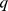
’ on the path (e.g., 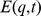).
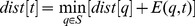
(1)


### 0.4 Structural separation of vessel trees

Dijkstra's algorithm determines the minimum edge cost path (smallest sum of edge costs on the path) ‘dist[e]’ from seed node ‘
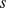
’ to each of the end point nodes ‘
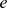
’ representing vessel end point segments (marked red in (Fig. 4(b))), by selecting the intermediate nodes ‘
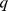
’ which minimize the sum of edge costs on the path (Eqn. 2), i.e., the intermediate vessel segments which minimize the cost differences along the path. The value of ‘dist[e]’ for each ‘
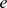
’ is normalized by the number of nodes (vessel segments) on the path, given as ‘
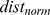
[e]’.
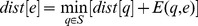
(2)


**Figure 6:**
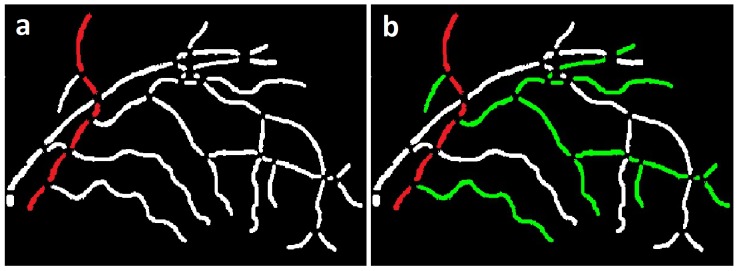
Image example: a) Vessel segment map showing the true vessel path b) True vessel path with branches.

The theoretical assumption is that the true vessel path is governed by the lowest edge cost path ‘
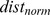
[e′]’ among all the edge cost paths ‘
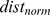
[e]’ determined for the respective end point nodes ‘
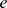
’ (Eqn. 3). In other words, the path with lowest sum of edge costs ‘
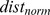
[e′]’ along the total path length, from seed node ‘
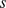
’ to one of the end point nodes (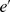), would be the path with least cost differences between the vessel segments, i.e., most similar segments on the path (segments marked in red (Fig. 6(a))), comprising a true vessel. The pseudocode for the structural mapping using Dijkstra's graph search is given in algorithm in Table 1.
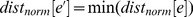
(3)


**Figure 7:**
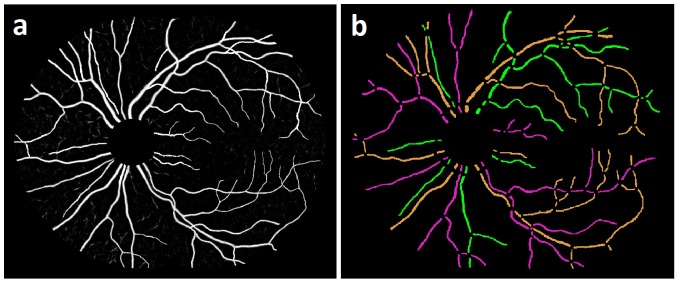
Image example: a) Vessel probability image b) Structural mapping of vessel network.

Utilizing the branch and crossing point information (Fig. 4(b)) along with the selected true vessel path, the branches and sub-branches are determined using the same principle, as above. A true or primary vessel path (marked in red), the branches and the sub-branches (marked in green) in a vessel tree are mapped as shown in Figure (Fig. 6(b)). The primary vessel, its branches and sub-branches in each vessel tree may be identified by numerical or color labels as shown in (Fig. 7(b)).

### 0.5 Identification of artery-venous crossing and color properties specific to arteries and veins

**Figure 8:**
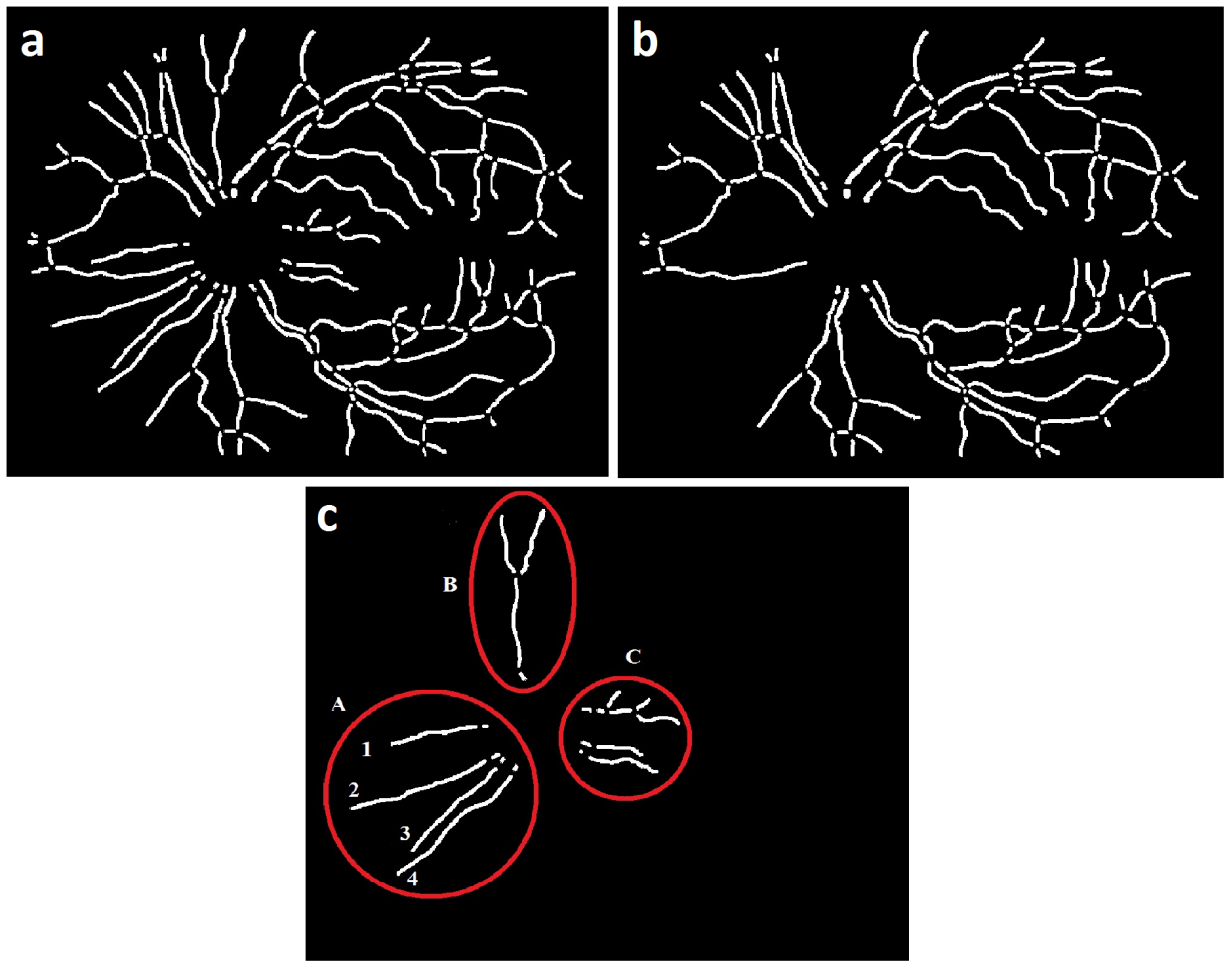
Image example: a) Vessel segment map b) Vessel trees with AV crossing c) Vessel trees without AV crossing.

We propose an automated AV separation algorithm based on structural mapping, which classifies the vessel trees into arteries and veins, using vessel color features as well as the anatomic property of artery-venous (AV) crossings. This property constrains that in the normal retina, the crossing of two retinal blood vessels in a two-dimensional fundus image, means that one is an artery and the other one a vein. In other words, at a vessel crossing, there is a low probability of both vessels being of the same kind; i.e., both arteries or both veins. Therefore, as an initial task, the vessel trees are separated into those with (Fig. 8(b)), and those without (Fig. 8(c)) arterial-venous crossing. The vessel segments (Fig. 8(a)) are skeletonized to obtain the vessel centerlines. For the centerline extraction, significantly large vessel width segments in a vessel tree are selected to avoid the inclusion of smaller, peripheral or single pixel width segments. It may prevent the effect of noisy centerlines on color feature extraction. A significantly large vessel width is defined for a particular vessel tree locally, and is determined as the width more than 60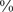 of the maximum vessel width obtained in that vessel tree.

A feature vector consisting of four features, viz., mean (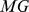) and standard deviation (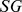) of green channel (from RGB color space) (Fig. 2(b)), and mean (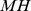) and standard deviation (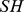) of hue channel (from HSV color space) (Fig. 2(c)), from the 3×3 neighborhood (region of interest) of each vessel centerline pixel is acquired. The choice of particular color features has been shown to be capable of distinguishing between arteries and veins [3], [9]. Arteries appear brighter (higher green channel intensity: 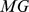) than veins because oxygenated hemoglobin is less absorbent than the de-oxygenated blood between 600–800 nm [19].

### 0.6 Arterial-Venous classification of retinal vessels based on fuzzy C-means clustering

**Figure 9:**
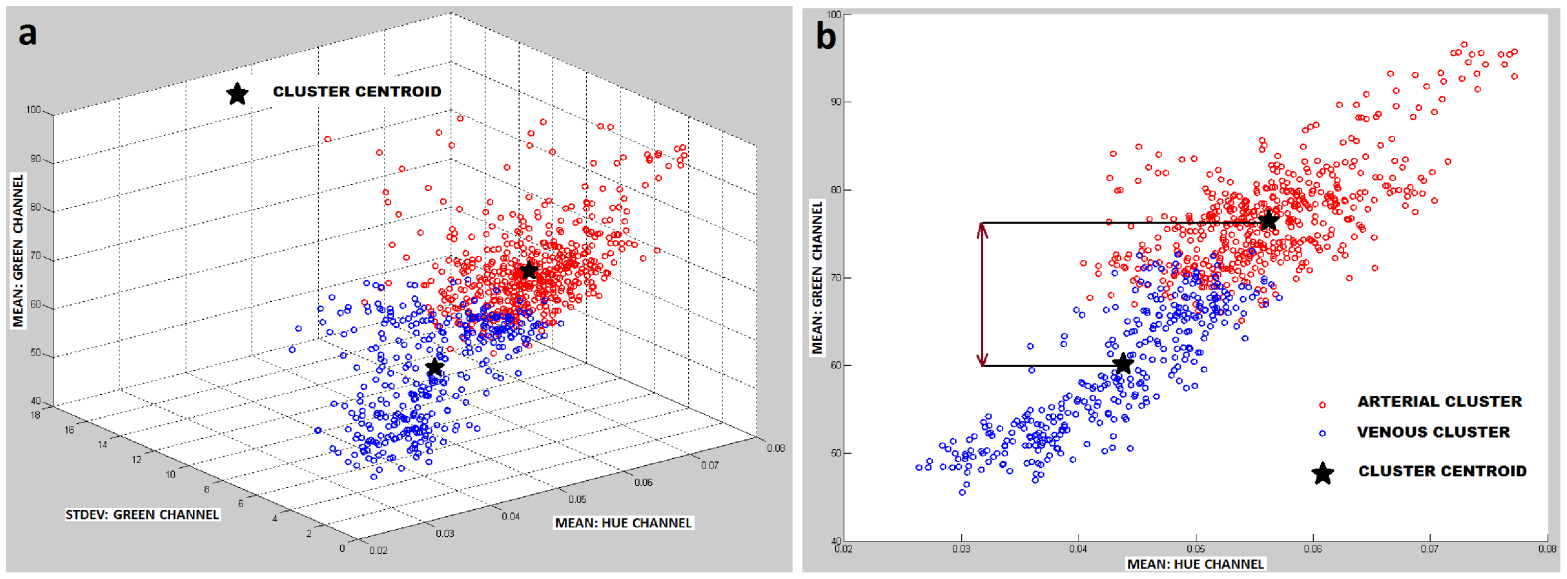
Fuzzy C-means clustering: a) Cluster formation b) Comparison of mean green channel intensity.

The centerline pixels obtained from any two vessel trees are collected and classified to detect the AV status of respective vessel trees. Based on the associated feature vector, the algorithm classifies the centerline pixels obtained from a pair of vessel trees, into two clusters/classes (with respective centroids) using the fuzzy C-means clustering algorithm. Each centerline pixel is assigned a degree of belonging to each of the two clusters (a number between 0–1), based on Euclidean distance measurement between the cluster centroid and the pixel in feature space. The two degrees assigned to a centerline pixel always sum to 1. A centerline pixel with difference between two degrees, higher than 0.2 (e.g., 0.39 vs. 0.61), is assigned to the higher degree cluster. The use of fuzzy C-means clustering helps eliminate the centerline pixels with difference between two degrees, of less than 0.2, i.e., having more or less equal affinity (e.g., 0.45 vs. 0.55) towards both clusters. These indeterminate pixels are treated as noise and are removed from further analysis. Fig. 9(a) shows the formation of two clusters in a three-dimensional view with axes represented by 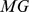, 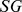, and 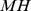, and centroids marked with a black star (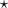) symbol. The centroid of each of the two clusters is a co-ordinated vector of average values of 4 feature properties associated with centerline pixels in that cluster [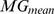, 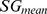, 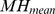, 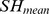]. The clusters are labeled as arterial or venous, based on the numerical comparison of averages of mean green channel intensity (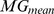) of two centroids. Fig. 9(b) shows the projection of clusters in (Fig. 9(a)) on a two-dimensional plane formed by 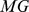 and 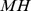, with 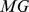 represented on Y-axis. The cluster with higher average value of mean green channel intensity (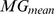) is labeled as arterial cluster and the other cluster as the venous cluster, since arteries appear brighter relative to veins.

**Figure 10:**
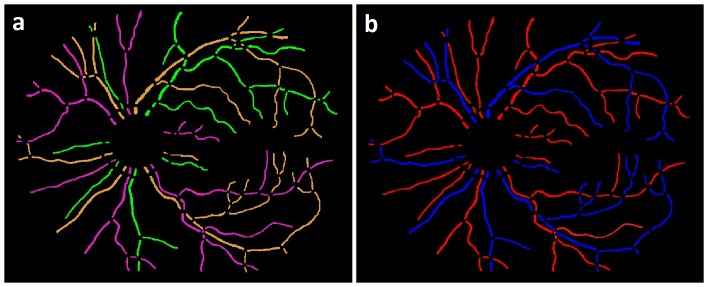
Image example: a) Structural mapping b) Artery-Venous Classification.

For a pair of vessel trees with AV crossings (for which we have a prior knowledge of them being of different types), the vessel tree with higher proportion of arterial class centerline pixels compared to the other vessel tree is labeled as an arterial tree, and the other one is labeled as a venous tree. For the vessel trees with no AV crossing (unpaired), we use the following method for their classification. Each of the unpaired vessel trees is designated to a group if the Euclidean distance between the centroid pixel (center of mass) of a given unpaired vessel tree and the centroid pixel of at least one unpaired vessel tree in that group is less than the predefined threshold, that indicates their spatial nearness. The threshold is defined empirically based on the image resolution. Thus, each such group of unpaired vessel trees in a localized region is analyzed separately which may prevent the non-uniform illumination effect on localized color feature extraction process, as shown in (Fig. 8(c)) for groups A, B, and C. The vessel trees in any one group at a time, are organized in pairs such that the mutual comparison is possible, e.g., Vessels 1,2,3 and 4 in group A are compared in pairs such as 1–2,1–3,1–4,2–3,2–4 and 3–4. If a group consists of only one single vessel (e.g., group B), this vessel is merged into spatially nearest group of vessels (e.g., group C) for analysis. For each pair of vessels, the most probable class of centerline pixels is identified for each vessel; i.e., the class pixels (arterial or venous) occupying in higher proportion of centerline pixels on that vessel. As the statuses of both the clusters/classes are already determined, each vessel in a pair is soft-labeled with the corresponding high probability class label. This procedure is followed for all the vessel pairs in a group and each vessel in a group is soft-labeled number of times depending upon the number of vessels in that group. A hard label is assigned to each vessel as the median value of all the soft labels received for that vessel. The vessels without AV crossing are classified based on the most probable class of centerline pixels but with mutual comparison between vessel pairs and a voting procedure (i.e., median of soft labels). The AV classification results are shown in (Fig. 10(b)), with arteries marked in red and veins marked in blue.

## Materials

We evaluated the dataset of 50 digital color fundus images of 50 subjects selected randomly from EYECHECK database. More information about the dataset can be found elsewhere [20]. The dataset consists of standard two-field registered fundus images (768×512, 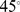 FOV) from subjects with diabetes with and without diabetic retinopathy. The images were deidentified before being accessible to the authors of this study, and thus this study was not human subjects research. The proposed method was applied to the dataset and the images used to design and implement the algorithm were excluded from the evaluation data. The fundus images were processed to obtain the vessel segmentation and the vessel segment map as shown in (Fig. 8(a)). In order to validate the structural mapping and the AV classification produced by the automated method, the vessel segment map (Fig. 8(a)) was annotated manually by a trained human grader using color labels for structural mapping, whereas red (artery) and blue (veins) labels for AV classification. We used previously validated Java based Truthseeker desktop application for expert annotation of vessel trees [21].

## Results

A copy of the vessel segment map (as above) was also labeled using the automated method by preserving the respective color code followed by the grader. To evaluate the accuracy of the proposed method, the automated labeling was compared with the expert annotation in terms of a segment color value. A segment marked with equal color value by both automated method and expert annotation was treated as accurately classified segment, and vice versa.

**Figure 11:**
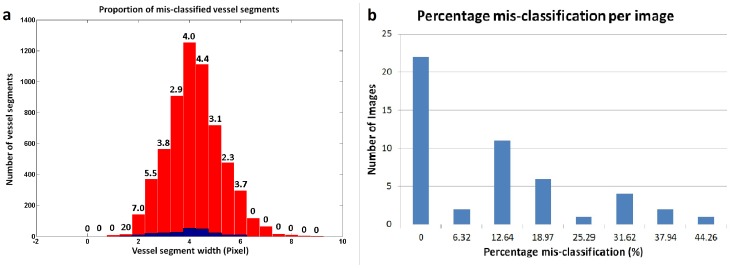
Metrics: a) Proportion of mis-classified vessel segments, b) Percentage mis-classification per image.

Two metrics were utilized to quantify the accuracy of the method. The first metric calculates the mis-classification rate (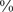) for vessel segments as a function of vessel segment width, over the dataset (Fig. 11(a)). The red bar in the histogram shows the total number of vessel segments (Y-axis) within a particular width interval (X-axis), whereas the respective blue bar shows the number of mis-classified vessel segments in the same interval. The number shown on top of each red bar represents the mis-classification rate (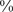) for vessel segments within that width interval. The mis-classification rates (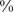) for various vessel segment sizes were categorized in Table 2. The average mis-classification rate (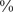) for vessels with width above 4 pixels was 3.58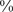. Thus, given a randomly selected medium sized or major retinal vessel, it would be classified correctly in 96.42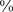 of cases.

**Table 2:** Proportion of mis-classified vessel segments.

Vessel size	Vessel width (Pixel)	Vessel segment mis-classification (%)
Small/Peripheral	1≤width<4	4.07
Medium	4≤width≤6	3.78
Major	6<width≤9	0.00

The second metric (Fig. 11(b)) shows the histogram of pixel mis-classification (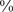) per image, in the dataset. The Y-axis shows the number of images for which the pixel mis-classification (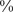) was within the interval represented on (X-axis). For each image, the pixel mis-classification (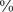) was calculated as the fraction of total number of vessel pixels which was mis-classified, representing its impact on the vessel network. The average mis-classification of 8.56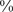 or the accuracy of 91.44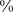 correctly classified vessel pixels was obtained over the dataset.

The average mis-classification rate (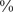) for single vessel trees (without AV crossing) was obtained as 17.07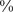, whereas the average mis-classification rate (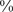) for paired vessel trees (with AV crossing) was determined as 4.96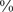. The difference between the mis-classification rates for single and paired vessel trees was statistically significant (p-value
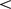
0.05).

**Figure 12:**
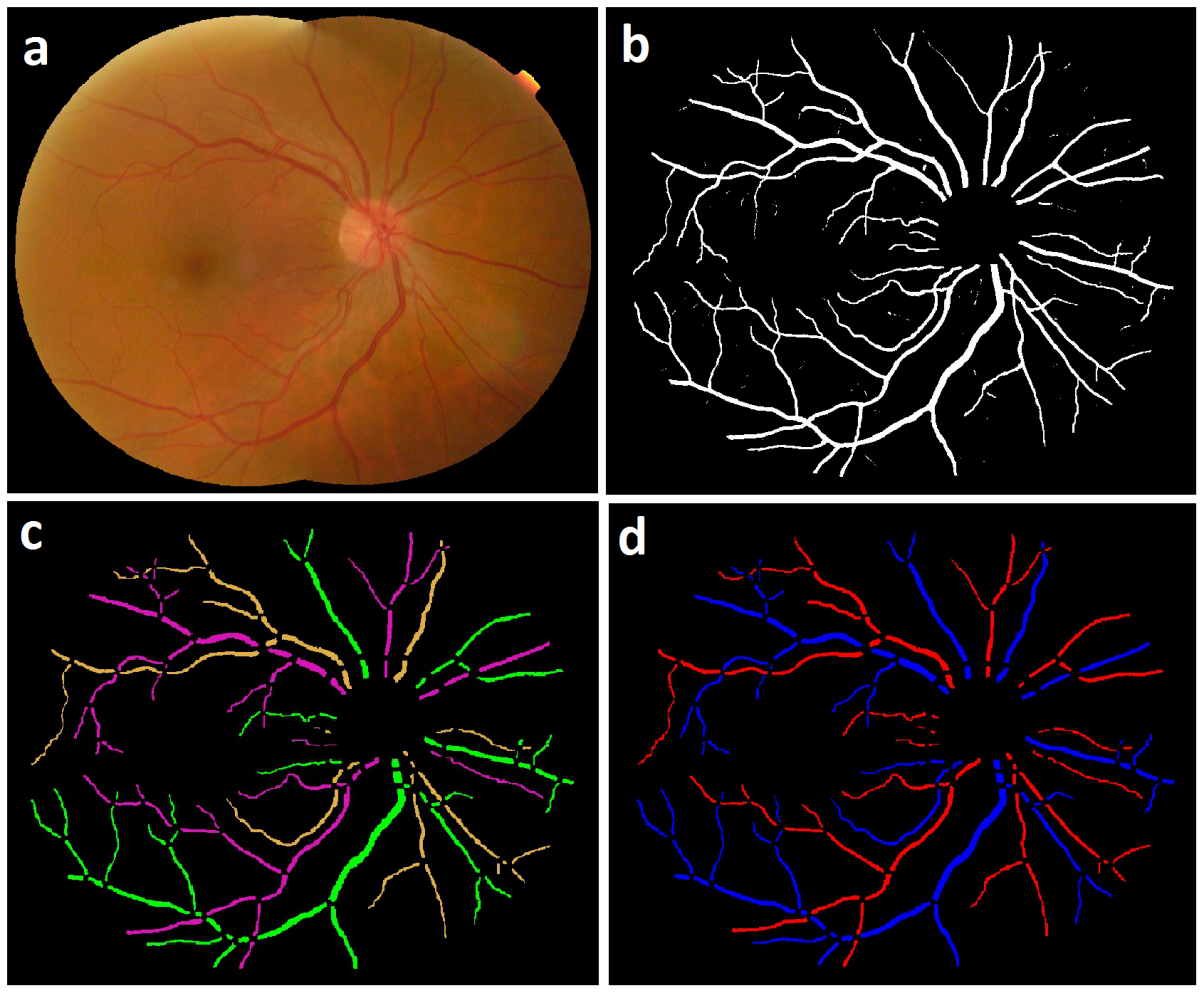
Image example: a) Fundus image b) Vessel probability image c) Structural mapping d) AV Classification.

The average running time per image starting at the readily available vessel segmentation to AV classification was 8 minutes including 7 minutes for structural mapping and 1 minute for subsequent AV classification, when processed in MatLab environment on a standard personal computer with Intel core 2 Duo processor, running at 3 GHz. The algorithm was not optimized for speed. The automated structural mapping and AV classification of retinal vessel trees is shown in Fig. 12.

## Discussion

We developed an automated method for identifying and separating the retinal vessel trees in color fundus images, which provides the mapping of primary vessels, and their branches. The strategy of modeling the vessel segmentation into vessel segments, characterizing their properties, i.e., orientation, width and intensity, and minimizing the difference between these properties to identify a true vessel, may work well for structural mapping. Furthermore, we described the mapped vessel trees in terms of arteries and veins.

**Figure 13:**
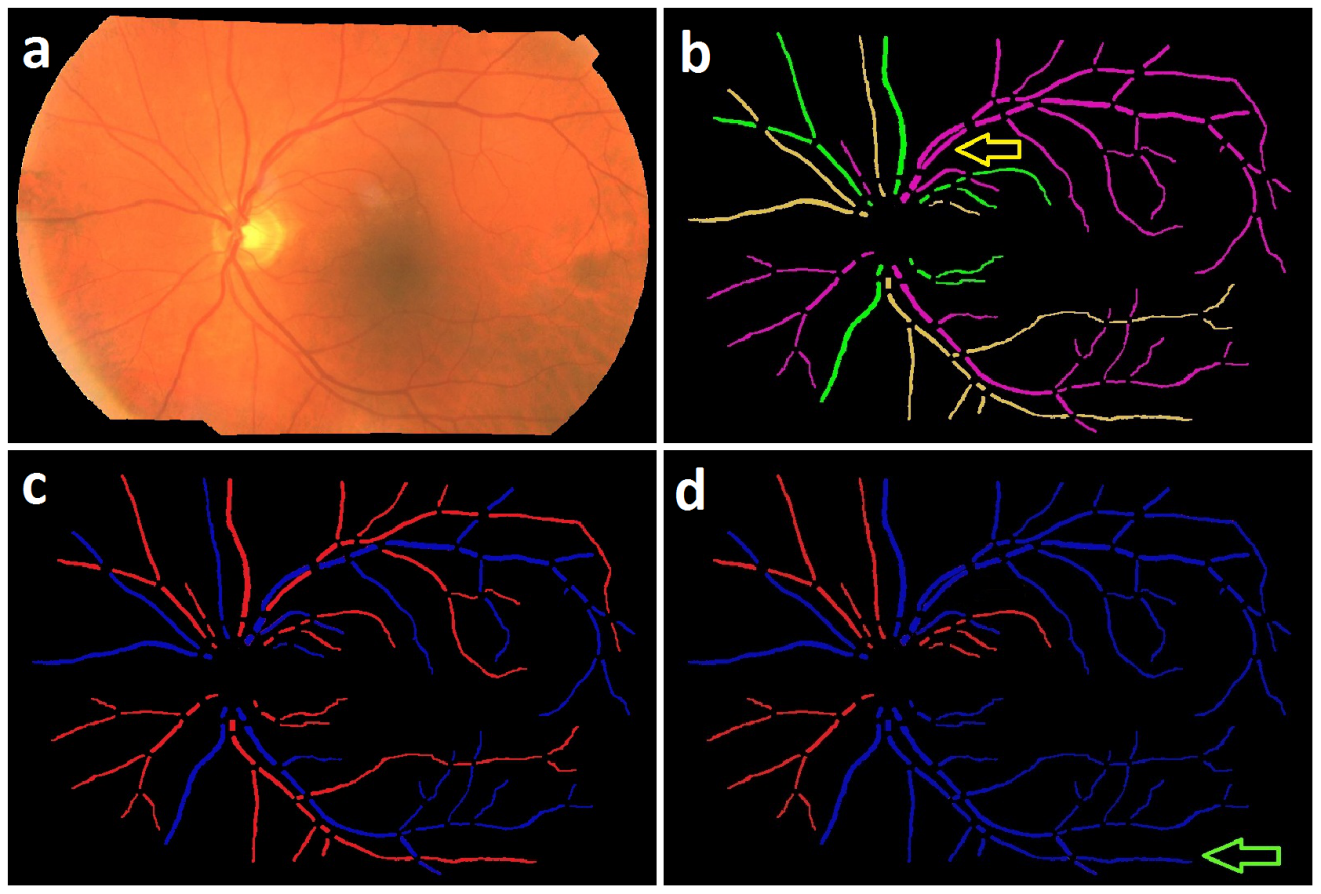
Image example: a) Fundus image b) Structural mapping c) Manual AV labeling d) Automated AV Classification.

Our results demonstrate that an automated method is capable of separating and classifying retinal vessel trees with an accuracy comparable to that of experts. The first metric reports the average mis-classification rate below 5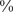 for vessel segments. This mis-classification rate decreases further to 3.58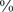 if only medium sized and major vessels are considered, as a clinician may find their diagnostic importance higher compared to smaller or peripheral vessels. Therefore, the diagnostically relevant vessels may be classified correctly 96.42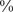 of the times. The second metric provides the average accuracy of 91.44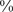 correctly classified vessel pixels (vessel network area), and enables the determination of the overall impact of mis-classification on the vessel network. The results (Fig. 11(b)) show six outliers representing images with more than one-third of the vessel region classified falsely. The image with highest mis-classification of 44.26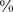 is shown in (Fig. 13), which was partially contributed by both false structural mapping and false AV classification, as will be discussed later. On an average, the proposed method may be capable of classifying at least 90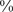 of the vessel network accurately.

The structurally separated vessel trees were classified using color properties that distinguish between an artery and a vein. The classification based on localized color features along with mutual comparisons and a voting procedure may have reduced the effect of intensity variations across the image, and across different subjects. The artery-venous crossing property determined the vessel pairs with a high probability of vessels being of different types, and enhanced the classification performance. It is evident from the smaller contribution of paired vessel trees (4.96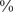) in mis-classification relative to that of single vessels (17.07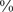), and statistically significant difference between the mis-classification rates for both vessel types.

The AV classification methods [3], [9] reported previously, depend upon the color features of vessels to discern between an artery and a vein. For feature extraction, the vessel segments are selected from a definite region of interest (ROI), which may exclude the posterior pole as well as the peripheral retina. The region selection constraint may reduce the strength of a feature set, and may limit AV classification only to the vessels inside the ROI, which restricts the measurement of parameters such as AVR to a limited region. The propagation of AV classification inside of ROI to the periphery may be complex due to the factors such as the AV crossings where artery and vein may run parallel to each other, an AV crossing superimposed on a vessel branching, ambiguous vessel connections due to tortuous or small vessels, multiple wriggling of two vessels upon each other, or multiple AV crossings between 2 vessels that produce cycles when imaged on a two-dimensional fundus image. Rothaus et al. [7] discuss variety of such vascular interactions due to which the propagation of AV classification to the outside of the ROI becomes complex and requires a rule-based approach. The methods [3], [9] classify vessels only inside the ROI where the aforementioned complications may be less severe, but their propagation to the outside of the ROI may be difficult without the knowledge of structural properties of vessels. Therefore, it may be imperative to consider vascular features such as orientation, width, and intensity; and principles of vascular tree connectivity, branching and tapering; to identify a true vessel and to propagate AV classification to the periphery, as described in [4], [7], [22], [23]. The proposed method provides a structural mapping for vessel tree identification that enables AV feature extraction without the ROI constraint and AV classification over the complete vessel network.

The proposed method provides the separation of vessel trees into arteries and veins as well as into primary vessels, and their branches, which may reduce the intertwining complexity of the retinal vessel structure that normally prevents the accurate measurement of individual vessel properties. This analysis may enable the automated measurement of morphologic parameters including branching angle and branching coefficient which change during the development of diabetic retinopathy [24], [25]. An added advantage may be the inclusion of smaller and peripheral vessels into the measurement system without following the constraint over the vessel size as in [26], or the specified ROI of the fundus as in [27].

As per our knowledge, the only method for retinal vessel identification and separation was proposed by Lau et al. [5]. This method, however, is restricted to the analysis of vessels inside a region of interest (ROI) around the optic disc, where the complications due to the vascular interactions as discussed in [7], may be less severe. As explained previously, the identified vessels therefore cannot be propagated outside the ROI without a rule-based approach. This limits it's practical utility for multi-field images mosaiced together. This method presents a pixel based accuracy of approximately 98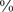 for clean segmented images [5], which is comparable to the pixel based accuracy of 91.44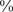 for vessel identification and AV classification as obtained by our method (Second metric), recognizing that our results are not limited only to the clean segmented images or only to the vessels inside of ROI. Furthermore, the pixel based accuracy may be biased by the pixel contribution from large sized (width) vessels, and therefore our results account also for the accuracy by vessel sizes (First metric). The first metric shows equally comparable accuracy of 96.42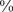 in identification and classification of diagnostically important medium and major sized vessels. One step ahead of the vessel separation and identification, our method also provides an automated means of classifying separated vessel trees into arteries and veins, which is currently unavailable in Lau et al.'s approach. The performance of the method by Lau et al. may be compromised by the presence of interrupted vessel segmentation, whereas our method provides a supporting algorithm with a fully automated procedure for reconnection of vessel interruptions to produce a clean segmentation [14].

The method reported by Aylward et al. identifies and separates the intra-cranial vessels imaged in three dimensional CT and MRA images [6]. Although not directly related to the identification of retinal vessels, however, unlike to this method, our proposed method works with vessels imaged in two dimensional fundus images and utilizes the structural properties of vessel segments to recover for the unavailability of third dimensional data. Few other methods developed for retinal vessel tracking in two dimensions [28], [29], may track the vessel without a control over its individual structural propagation. Therefore, they may not provide the identification and the separation of individual vessel trees. To the best of our knowledge, the proposed method for structural mapping and AV classification of the entire retinal vessel network imaged in two-dimensional color fundus images is novel.

Some limitations of this method are the following. The method requires high quality connected vessel segmentation image for the structural mapping. Therefore, lower quality segmentation including interrupted vessels may result in errors. We previously developed a method for identifying and reconnecting the interrupted vessels using graph search which may be able to provide a connected vessel structure [14]. The other limitation is its inability to identify and separate two vessels overlapping (due to two dimensional imaging) or touching each other in a parallel course (Fig. 13(b):Yellow arrow), which may be improved using methods presented elsewhere [30]. The AV classification method is based on color features of vessels and therefore limited by the non-uniform illumination effects and low contrast in the fundus image. These may result into a false classification of arteries and veins due to the localized illumination effect. Fig. 13(c) shows the AV classification by a trained grader, and Fig. 13(d) shows the automated AV classification where artery in a local darker region is classified as a vein (Green arrow).

A preliminary version of this work appeared in SPIE conference proceedings [31] and [32]. As presented in the preliminary research articles, the structural mapping algorithm when applied individually to the set of 15 fundus images resulted into an accuracy of vessel separation of 92.87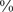, whereas the subsequent application of artery-venous (AV) classification algorithm to the same set of images resulted into an accuracy of 88.28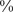. The manual analysis of these results indicated that the majority of the total misclassification was introduced due to the limitations in structural mapping and was supplemented further due to the limitations in AV classification. The quality of AV classification depends upon the quality of vessel separation by structural mapping up to a certain extent. The limitations of structural mapping method such as in separating the overlapping or parallel running vessels or in resolving the complications due to vascular interactions as discussed in [7], result into false vessel identification and therefore a false AV classification. However, since the AV classification process depends upon the localized color feature analysis and the property of AV crossing, it is relatively robust to the structural mapping errors localized to a certain region and prevents it's effect on the classification of rest of the vessel network. The accuracy of AV classification is dependent upon the accuracy of the preceding step, i.e. structural mapping, and the error introduced during the structural mapping process may not be resolved and/or corrected entirely as a part of the final classification output. A different vessel separation method with a higher accuracy, e.g., [4] (applicable to fluorescence angiography images), introduced as the basis to the AV classification process may report a superior performance.

In summary, we developed an automated method for identification and AV classification of retinal vessel trees in fundus color images. The properties of a vessel structure, i.e., orientation, width and intensity, were utilized to identify the vessel tree, and its color as well as crossing properties classified it as an arterial or a venous vessel tree. The proposed method was validated on a fundus color image dataset showing results that match well with the expert annotations.

## Conclusion

The research presents a novel method for identification and AV classification of retinal vessel trees in color fundus images. A fundus image and the corresponding vessel segmentation image are processed to obtain the separation of intertwined vessel trees, and their description in terms of arteries and veins. The structural mapping and the AV classification results match well with the expert annotations. This approach has the potential to impact the diagnostically important morphologic analysis of individual retinal vessels.
